# Reversible phosphorylation of the 26S proteasome

**DOI:** 10.1007/s13238-017-0382-x

**Published:** 2017-03-03

**Authors:** Xing Guo, Xiuliang Huang, Mark J. Chen

**Affiliations:** 1 The Life Sciences Institute of Zhejiang University, Hangzhou, 310058 China; 2 Ministry of Education Key Laboratory of Protein Science, School of Life Sciences, Tsinghua University, Beijing, 100084 China; 3 Department of Bioinformatics and Computational Biology, Genentech Inc., South San Francisco, CA 94080 USA

**Keywords:** proteasome, phosphorylation, kinase, phosphatase, protein degradation

## Abstract

The 26S proteasome at the center of the ubiquitin-proteasome system (UPS) is essential for virtually all cellular processes of eukaryotes. A common misconception about the proteasome is that, once made, it remains as a static and uniform complex with spontaneous and constitutive activity for protein degradation. Recent discoveries have provided compelling evidence to support the exact opposite insomuch as the 26S proteasome undergoes dynamic and reversible phosphorylation under a variety of physiopathological conditions. In this review, we summarize the history and current understanding of proteasome phosphorylation, and advocate the idea of targeting proteasome kinases/phosphatases as a new strategy for clinical interventions of several human diseases.

**Electronic supplementary material:**

The online version of this article (doi:10.1007/s13238-017-0382-x) contains supplementary material, which is available to authorized users.

## INTRODUCTION

The year of 2017 marks the 30th anniversary of the discovery of proteasome, the central hub of protein degradation in all eukaryotic cells (Hough et al., 1987; Waxman et al., 1987). The past three decades have witnessed enormous advancement of our understanding about proteasomal degradation of proteins involved in almost every aspect of cell biology. The biological importance, biochemical complexity, and clinical relevance of the proteasome system are now well established, although many important details of proteasome function, structure, and regulation remain elusive and will continue to be topics of intensive research (Finley, 2009; Schmidt and Finley, 2014; Finley et al., 2016; Livneh et al., 2016).

The core of all proteasome complexes is a 28-subunit, barrel-shaped structure known as the 20S proteasome or core particle (20S CP). These subunits are arranged as four stacked rings (Groll et al., 1997; Unno et al., 2002). The two outer rings (at the top and bottom of the CP) are made of α subunits (α1–7, designated PSMAs in human and higher eukaryotes), whose N-termini form a “gate” at the axial center and occlude the entrance into the CP chamber. Each of the two inner rings is composed of subunits β1–7 (PSMBs). Three of the β subunits, namely β1, β2 and β5, function as threonine-proteases and preferentially cleave substrate polypepetides after acidic (caspase-like activity), basic (trypsin-like activity), and hydrophobic residues (chymotrypsin-like activity), respectively. All their N-terminal active sites are positioned at the interior center of the CP. In addition to these constitutive subunits, the CP can incorporate specialized subunits such as β1i, β2i, and β5i to form immunoproteasomes (Kloetzel, 2001), or β5t to form thymoproteasomes (Murata et al., 2007), or α4s instead of α4 in the testis (Uechi et al., 2014). Due to its unique architecture, the 20S proteasome in its free form cannot degrade folded protein substrates as they are inaccessible to the catalytic center.

For proteasomal degradation to occur, the gate formed by α subunits must be opened to allow for substrate entry. This “gate-opening” function can be achieved by several types of proteasome activators that directly bind the α ring, including the 19S regulatory particle (RP)/PA700, 11S/PA28/REG, and Blm10/PA200 (Stadtmueller and Hill, 2011). Thus, different forms of CP may associate with different activators, resulting in multiple types of proteasome complexes that co-exist in cells. The 19S RP has been widely studied and together with 20S CP forms the best known 26S proteasome, a 2.0–2.5 MDa machinery that degrades the vast majority of poly-ubiquitinated as well as some non-ubiquitinated proteins of the cell (Finley, 2009).

A total of nineteen subunits assemble into the 19S RP, including six AAA+ type ATPases (Rpt1–6, or PSMCs) and thirteen non-ATPase proteins (Rpn1–3, 5–13 and 15, known as PSMDs). Each Rpt subunit contains an N-terminal flexible region, a coiled-coil domain, an oligonucleotide-binding (OB) domain and an ATPase domain. The coiled-coil regions are required for dimerization of Rpt1-Rpt2, Rpt3-Rpt6, and Rpt4-Rpt5, which join with one another in the presence of multiple assembly chaperones to form a hexameric ATPase ring that directly caps one or both ends of the CP (Funakoshi et al., 2009; Kaneko et al., 2009; Murata et al., 2009; Park et al., 2009; Roelofs et al., 2009; Yu et al., 2010). In the Rpt ring structure, the OB and ATPase domains make up the central channel, which upon substrate polypeptide binding aligns with the CP gate to form a continuous passage. Substrate engagement with Rpts also stimulates their ATPase activity that in turn provides the necessary energy for substrate unfolding before its translocation to the CP (Smith et al., 2005; Peth et al., 2013). The extreme C-termini of Rpt2, 3, and 5 contain a HbYX motif (hydrophobic residue-tyrosine-any amino acid). They play critical roles in RP-CP interaction by directly inserting into pockets of the α ring, at the same time causing significant conformational changes and opening of the CP gate (Smith et al., 2007; Rabl et al., 2008; Park et al., 2009). The coiled-coil, OB, ATPase domains and the HbYX motif are well defined in crystal and cryo-EM structures, and their primary sequences are highly conserved through evolution (Djuranovic et al., 2009; Chen et al., 2016; Huang et al., 2016; Schweitzer et al., 2016). On the other hand, the extreme N-termini of Rpts appear to be poorly structured and less conserved, although they harbor modification sites that are important for modulating proteasome functions (See later).

Rpt1–6 and three non-ATPase subunits (Rpn1, 2, and 13) are traditionally referred to as the “base” of the 19S RP, while the remaining Rpn subunits constitute the “lid”. In the cyro-EM models, Rpn2 is positioned at the apex of the 26S holoenzyme (farthest from the 20S CP) and directly contacts the coiled-coils of Rpt3-Rpt6. The latter serves as a pivot around which the lid complex rotates in accord with substrate engagement, unfolding, and translocation (Matyskiela et al., 2013; Unverdorben et al., 2014). Rpn1, Rpn10, and Rpn13 function as receptors for ubiquitin and ubiquitin-like (UBL) domain proteins (Deveraux et al., 1995; Husnjak et al., 2008; Schreiner et al., 2008; Shi et al., 2016). The proteasome-intrinsic de-ubiquitinating enzyme Rpn11 and its partner Rpn8 cleave off ubiquitin chains from committed protein substrates in order to facilitate substrate unfolding, translocation, and ubiquitin recycling (Verma et al., 2002; Yao and Cohen, 2002; Worden et al., 2014). The rest of RP subunits (Rpn3, 5, 6, 7, 9, 12, 15) are not known to possess enzymatic or receptor properties but play essential structural functions in 26S proteasome assembly. Working as a complex, the 19S RP is responsible for (i) substrate recognition and engagement, (ii) substrate de-ubiquitination, (iii) substrate unfolding and translocation, and (iv) 20S gate opening and activation. Of note, all these activities except for substrate recognition depend on ATP binding/hydrolysis by the ATPase subunits. Therefore, Rpt1–6 play structural, enzymatic, and regulatory roles that are essential for 26S proteasome function (Finley, 2009; Ehlinger and Walters, 2013).

The assembly of individual subunits into a functional proteasome is controlled by a series of chaperone proteins, representing the best characterized aspect of proteasome regulation (Murata et al., 2009). Most chaperones are absent/dislodged from the fully assembled complex, while dozens to hundreds of other cellular proteins can dynamically interact with the mature proteasome (Wang et al., 2007; Wang and Huang, 2008). Although the biological meanings of these interactions are largely unknown, many proteasome-interacting proteins (PIPs) have enzymatic activities and modify the proteasome in a variety of ways (reviewed by Scruggs et al., 2012; Cui et al., 2014). Not surprisingly, phosphorylation is one of the most frequent and better studied means of post-translational modification of the proteasome.

**Figure 1:**
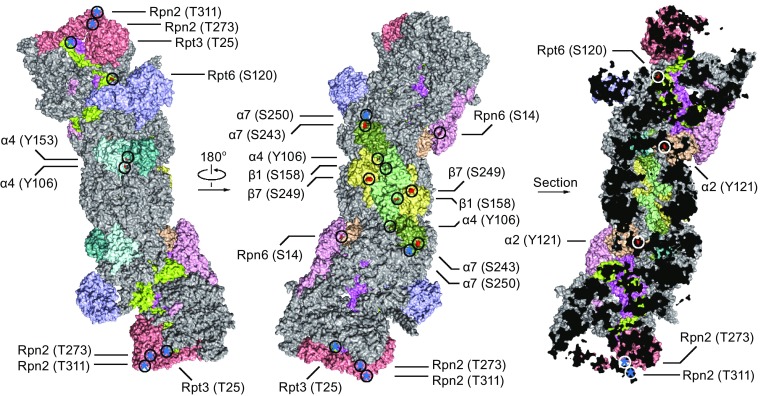
**Structural view of a selection of human 26S proteasome phosphosites**. Surface (left, middle) and sectional (right) views of human 26S proteasome (gray) are shown based on high-resolution cryo-EM structures (PDB 5GJR). Phosphosites of subunits Rpt6 (lemon), Rpn6 (light pink), α7 (smudge), Rpt3 (violet), Rpn2 (salmon), α4 (pale cyan), α2 (wheat), β7 (pale yellow), β1 (pale green) are marked with circles. Sites visible from available structures are highlighted in red, while predicted positions of invisible sites are represented with a blue star

In this review, we summarize our current understanding of proteasome regulation by reversible phosphorylation. Due to space limit, we only focus on phosphorylations of integral subunits of the constitutive human 26S proteasome (We will adhere to the nomenclature of α1–7, β1–7, Rpt1–6, and Rpns to avoid confusion) and highlight the functions of selected kinases/phosphatases and phosphosites (Fig. 1). We also discuss technical issues and potential clinical applications of present research on proteasome phosphoregulation.

## OVERVIEW OF 26S PROTEASOME PHOSPHORYLATION

The first documentation of proteasome phosphorylation dates back to 1989, not long after the discovery of the proteasome itself, when Haass and Kloetzel reported that proteasome subunits were modified (phosphorylated) in Drosophila cells. These researchers insightfully argued that “the *in vivo* proteolytic activity and the *in vivo* substrate specificity of the proteasome may be regulated by modification of its subunit composition during fly development” (Haass and Kloetzel, 1989). Their observations have now been supported by finer proteomic studies of many organisms, and yet the biological significance of proteasome phosphorylation during development is, by and large, still a mystery.

In the following decade, numerous independent reports had demonstrated phosphorylations of different proteasome subunits with limited information on their function. Phosphorylation was even once considered to partly account for the ATP-dependence of proteasome function (Ludemann et al., 1993). Some of the early studies reached inconsistent and occasionally contradictory conclusions as to which subunits were phosphorylated on which sites, largely due to differences among cell types (with distinct expression and activity of kinases/phosphatases), species (with or without a non-conserved phosphosite), and purification procedures (Labile phosphorylations are easily lost during lengthy chromatography or in the absence of phosphatase inhibitors). Moreover, it was very difficult, if not entirely impossible, to determine the exact phosphorylation sites of a gigantic protein complex like the proteasome simply by ^32^P labeling, 2D-electrophoresis, and phospho-amino acid mapping. In fact, phosphorylations detected by these methods were either highly abundant in a particular cell/tissue type, or fairly resistant to dephosphorylation, or possibly artifacts of proteasome purification and *in vitro* kinase reactions.

The advent of tandem mass spectrometry (MS/MS)-based phosphoproteomics caused revolutionary changes in our understanding of proteasome regulation. Less than a dozen phosphosites were known to exist on human 26S proteasome by the early 2000’s, while the number rocketed to over 300 by 2013 according to PhosphoSitePlus, one of the most comprehensive phosphoproteomic databases (www.phosphosite.org). The current tally (by July, 2016) is 455 phosphosites. These include 201 phospho-serine (pS) sites, 104 phospho-threonine (pT) sites, and 150 phospho-tyrosine (pY) sites, all but two of which have been detected by large-scale MS studies. A complete list of all human proteasome phosphosites is available in Table S1. In the following discussions, we will primarily rely on information from PhosphoSitePlus, and refer to the PhosphoGrid (phosphogrid.org) and PhosphoMouse (https://gygi.med.harvard.edu/phosphomouse/) databases for phosphorylations of yeast and mouse proteasomes, respectively.

Proteasome phosphorylations are seen in almost every large-scale phosphoproteomic dataset. More importantly, the proteasome is dynamically phosphorylated in a variety of physiological and pathological processes, including development and stem/progenitor cell differentiation (Brill et al., 2009; Rigbolt et al., 2011; Goswami et al., 2012), cell cycle (Beausoleil et al., 2006; Dephoure et al., 2008; Nagano et al., 2009; Olsen et al., 2010; Kettenbach et al., 2011; Guo et al., 2016), DNA damage response (Matsuoka et al., 2007; Stokes et al., 2007), stress responses (Um et al., 2010; Wang et al., 2014), immune signaling (Bose et al., 2001; Bose et al., 2004; Mayya et al., 2009; Weintz et al., 2010; Wu et al., 2012), metabolic changes (Bardag-Gorce et al., 2004; Zhang et al., 2007b; Trost et al., 2012), neuronal activity (Djakovic et al., 2009; Bingol et al., 2010; Djakovic et al., 2012; Hamilton et al., 2012; Jarome et al., 2013; Jarome et al., 2016; Li et al., 2016), hormones and growth factor signaling (Kim et al., 2009; Pan et al., 2009; Lundby et al., 2013; Williams et al., 2016), and oncogenesis (Rush et al., 2005; Rikova et al., 2007; Guo et al., 2008; Luo et al., 2008; Choudhary et al., 2009; Eang et al., 2009; Iliuk et al., 2010; Johnson et al., 2012; Trost et al., 2012; Yuan et al., 2013). Although the functional roles of proteasome phosphorylation in these processes are largely uncharacterized, increasing evidence indicates that the 26S proteasome is not a uniform and static complex acting passively as a “cellular trashcan”. Rather, the proteasome itself is fine-tuned by reversible phosphorylation in response to intra- and extra-cellular signals, which can be a prerequisite or feedback mechanism for a wide spectrum of cellular events that depend on proteasome function.

A quick examination of the human proteasome phosphorylation data shows that phosphosites have been found on every subunit. The largest subunit Rpn2 has the most phosphorylation sites (27) while the smallest subunit Rpn15 has only one. However, no correlation exists between the number of phosphosites and the size of protein for most of the subunits. Overall, it appears that the 20S CP and the 19S base subunits are more frequently phosphorylated than the 19S lid, when the total number of MS detections for each site (based on the high-throughput, i.e. “HTP” numbers from PhosphoSitePlus) is taken into account. It should be noted, though, that more than half of the proteasome phosphorylations were detected only once by MS, and only 20% of all human proteasome phosphosites were observed for more than 5 times in all the studies combined. On the other hand, the frequency of MS detection of a proteasome phosphosite does not directly translate into its stoichiometry or functional importance, due to vastly different sample sources, purification/enrichment methods, detection instruments, and search databases used by various groups. As illustrated later in detail, some of the functionally important phosphosites have only been observed in a temporally or spatially restricted manner, demonstrating the intricate nature of proteasome phosphoregulation.

Of the 455 known phosphosites on human 26S proteasome, 442 (97.1%) are conserved or semi-conserved (i.e. Ser/Thr substitutions) in mouse and rat proteasome subunits, and 391 sites (85.9% of total) are found in zebrafish. However, the degree of site conservation drops considerably to 63.3% in fruit fly (*D. melanogaster*) and less than 50% in worm (*C. elegans*) and yeast (*S. cerevisiae*). Yeast 26S proteasome has been shown to be phosphorylated at low stoichiometry (Wu et al., 2011). The majority of yeast proteasome phosphorylation sites (Kikuchi et al., 2010), if conserved, are rarely phosphorylated in mammals. These observations suggest that novel phosphosites emerged during evolution (especially in vertebrates) as new regulatory mechanisms of proteasome function, a general theme that has been proposed for phospho-signaling (Holt et al., 2009).

**Figure 2:**
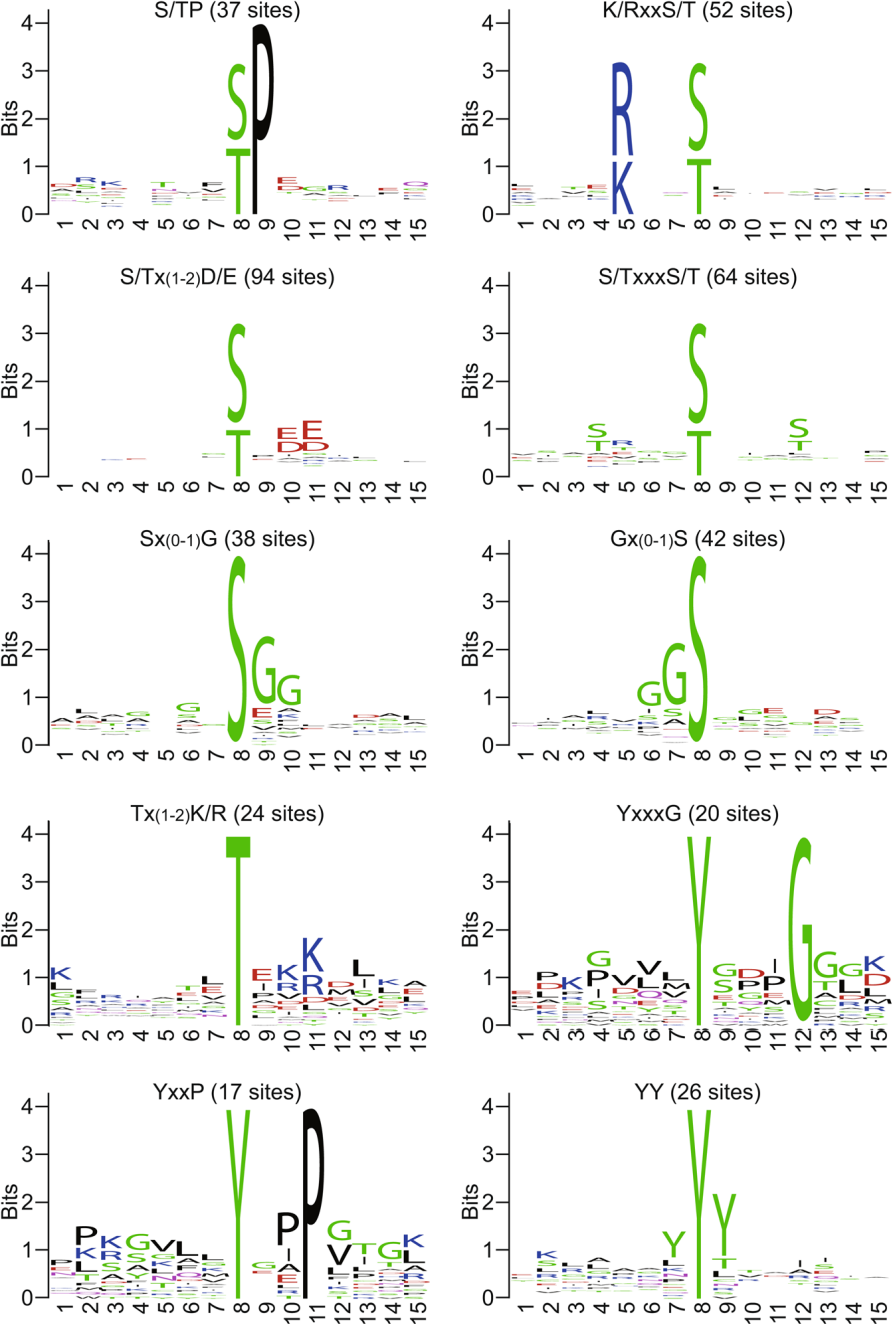
**Representative sequence motifs of human proteasome phosphosites**. All human proteasome phosphorylation sites were divided into pS, pT, and pY groups and their adjacent sequences were separately analysed with Motif-All. Similar motifs were combined and sequence logos of the ten most abundant representatives are shown. The X-axis shows amino acid positions with the phosphosites in the center. The Y-axis is the information content, which takes into account the frequency of amino acid across the proteome (background frequency) and is therefore a better measure

The surrounding sequences of many proteasome phosphosites conform to well defined recognition motifs of kinases, such as S/TP (MAPKs and CDKs), R/KxxS/T (AGC and CaMK families), S/TxxD/E (CK1 and CK2), and S/TxxxS/T (GSK3). Some of the most frequent motifs found on the proteasome are summarized in Figs. 2 and S1. Even with this information, it is generally a challenging task to pinpoint the kinase(s) for a given site, which has been a major hurdle toward understanding the regulation and function of proteasome phosphorylation. Up next, we will focus on functionally characterized proteasome kinases and phosphatases to showcase the biological relevance of proteasome phosphorylation.

## PROTEASOME KINASES AND PHOSPHATASES

### PKA

Protein kinase A (PKA) was probably the first kinase implicated in proteasome phosphorylation. The initial hint was co-purification of a cAMP-dependent kinase activity with the proteasome complex from bovine pituitaries. This putative kinase reportedly phosphorylated two proteins of 27–28 kDa, likely to be 20S subunits (Pereira and Wilk, 1990). Supporting the involvement of PKA in proteasome regulation, Marambaud et al. showed that the common activator of PKA, forskolin, stimulated endogenous proteasome activity in HEK293 cells. Purified PKA phosphorylated some 28–30 kDa subunits *in vitro*, leading to evident upregulation of peptidase activity (Marambaud et al., 1996). These original findings have been substantiated by subsequent studies. In particular, endogenous 20S proteasomes isolated from murine hearts also contains PKA α (catalytic) subunit as confirmed by Western blot and MS, and PKA-mediated *in vitro* phosphorylation enhances peptidase activities of 20S proteasomes isolated from both heart and liver (Zong et al., 2006; Lu et al., 2008). Interestingly, Zong et al. also indicated the presence of phosphatase PP2A (including its α, β, and γ subunits) in cardiac 20S complexes, and inhibition of PP2A by okadaic acid (OA) increased proteasome activity. These data demonstrate that phosphorylation by PKA positively regulates the 20S proteasome.

However, when incubated with purified intact 26S proteasome *in vitro*, PKA preferentially phosphorylates certain 19S subunit(s) instead of the 20S component (Zhang et al., 2007a and our unpublished observation). Just like the 20S CP, the 26S holoenzyme becomes more active upon PKA-mediated phosphorylation, an effect that can be reversed by the phosphatase PP1γ (Zhang et al., 2007a). MS analysis showed that recombinant PKA directly phosphorylates Rpt6 at Ser120 *in vitro*, while the Ser120Ala mutation blocks this phosphorylation and significantly reduces proteasome activity in cells (Zhang et al., 2007a). Echoing these results, Lin et al. reported that disease-causing mutants of Huntingtin (mHTT) could reduce overall PKA activity, Rpt6-S120 phosphorylation hence proteasome activity, causing a positive feedback leading to mHTT accumulation (Lin et al., 2013).

Rpt6-S120 is conserved from yeast to human and is arguably the best characterized among all proteasome phosphorylation sites, and yet several studies have convincingly demonstrated that it is in fact targeted by a different kinase, CaMKIIα (See later for details). Moreover, two groups have directly challenged PKA as the true Rpt6-S120 kinase (Jarome et al., 2013; Lokireddy et al., 2015). In fact, Ser120 of Rpt6 is preceded by an arginine residue at the −3 position (R_117_NDS_120_), which constitutes a typical RXXS/T recognition motif shared by AGC kinases (such as PKA) and CaMKs (such as CaMKIIα). It is possible that PKA phosphorylates Ser120 *in vitro* but not necessarily *in vivo*. In addition, detection of phospho-S120 with a phospho-specific antibody against PKA substrates (recognizing the RXXpS/T motif, Lin et al., 2013) does not establish PKA as the kinase. Moreover, PKA promotes the association between 19S and 20 proteasomes (See below), while S120 phosphorylation by CaMKIIα was shown not to affect proteasome assembly (Djakovic et al., 2009). Finally, in contrast to the wide distribution of PKA in many cell types, detection of endogenous Rpt6-S120 phosphorylation has primarily been restricted to neuronal cells, where CaMKIIα is highly enriched. Therefore, it remains to be determined whether PKA phosphorylates Rpt6-S120 *in vivo* at all, or if so, under what circumstances.

Nonetheless, PKA-mediated 26S proteasome activation and the ensuing beneficial effects on protecting neuronal cells from toxic protein aggregates have been established (Myeku et al., 2012; Lokireddy et al., 2015; Myeku et al., 2016). cAMP signal not only activates PKA but also increases the association between its catalytic subunit and the proteasome. This leads to phosphorylation of Rpn6 (but not Rpt6) at Ser14, shown by Phos-tag SDS/PAGE and confirmed by MS. Rpn6-S14 phosphorylation enhances proteasome ATPase activity and promotes the formation of doubly capped (30S) proteasome, hence accelerating the degradation of short-lived proteins (Table 1). The phospho-mimetic mutant Rpn6-S14D facilitates the clearance of a variety of aggregation-prone proteins closely associated with neurological diseases, while the phospho-deficient mutant Rpn6-S14A does the opposite (Lokireddy et al., 2015). Importantly, the effect of PKA activation on proteasome assembly occurs *in vivo* (Asai et al., 2009; Myeku et al., 2016). The latter study showed that rolipram (a specific phosphodiesterase type 4 inhibitor and PKA stimulator) activates proteasome in mouse brain and prevents proteasome impairment by mutant tau at the early stage of tauopathy, leading to improved cognitive ability (Myeku et al., 2016). More recently, researchers have demonstrated that many hormones (e.g. epinephrine and glucagon) and physiological responses (such as exercise or fasting) that increase cAMP levels also activate the proteasomes and stimulate Rpn6-S14 phosphorylation as shown with a phospho-specific antibody (personal communication with A. L. Goldberg). These findings are of obvious clinical relevance and suggest new approaches to (re)activate the proteasome and halt neurodegeneration. However, cautions should be taken because Rpn6-S14 phosphorylation also appears to be upregulated in cancer cells (Gnad et al., 2013; Mertins et al., 2014) and during T cell activation (Ruperez et al., 2012), the consequences of which have not been investigated.

**Table 1:** **A selection of human proteasome phosphosites with site-specific information.**

Subunit	Site	Sequence	Function	Kinase	Regulatory cues	Position on proteasome*	Predicted mechanism	Antibody	Reference
Rpt6	S120	RVALRND**S**YTLHKIL	Increases proteasome activity and tethering with actin cytoskeleton	CaMKIIα (PKA?)	Neuronal activity	Top of the OB-ring, close to the C-terminal helix of Rpn3	Facilitates substrate unfolding and translocation	Yes	Djakovic et al., 2009, 2012; Bingol et al., 2010; Hamilton et al., 2012; Jarome et al., 2013, 2016; Zhang et al., 2007a; Lin et al., 2013
Rpn6	S14	VEFQRAQ**S**LLSTDRE	Increases proteasome activity	PKA	cAMP signaling	Close to distal tip of Rpn6-N terminus, facing (but not close to) the α-ring	Stabilizes RP-CP interaction	Yes^#^	Lokireddy et al., 2015
α7	S243 S250	E**S**LKEEDE**S**DDDNM	Regulates the level of 26S proteasome?	CK2?	Constitutive. (IFN-γ?)	Outer surface of the α-ring, close to Rpt4	Stabilizes RP-CP interaction	No	Mason et al., 1996; Castano et al., 1996; Bose et al., 2001, 2004; Gersch et al., 2015
Rpt3	T25	LSVSRPQ**T**GLSFLGP	Increases proteasome activity	DYRK2	Cell cycle	* N-terminal to the coiled-coil region, contacting Rpn2	Facilitates Rpn2-lid rotation to facilitate substrate degradation	Yes	Guo et al., 2016
Rpn2	T273	QNLRTVG**T**PIASVPG	Inhibits proteasome activity	p38	Osmotic stress	* Between the N-terminal helical domain and C-terminal toroid	Regulates the motion transfer of Rpn2-lid rotation	Yes	Lee et al., 2010b
α4	Y106	EDPVTVE**Y**ITRYIAS	Maintains α4 protein level	Abl/Arg	?	Buried in the cleft between α4/α5, very close to β5	Regulates CP integrity	No	Li et al., 2015
α4	Y153	QTDPSGT**Y**HAWKANA	Inhibits proteasome activity	Abl/Arg	?	Buried in the cleft between α4/α5, very close to α5	Regulates CP integrity	No	Liu et al., 2006
α2	Y121	VASVMQE**Y**TQSGGVR	Controls nuclear import of the proteasome?	?	?	Inner surface of the CP chamber	?	No	Benedict and Clawson, 1996
β7	S249	VEIEGPL**S**TETNWDI	?	?	Decreased in cancer cells?	Outer surface of the β-ring	?	Yes	Eang, et al., 2009
β1	S158	GMMVRQ**S**FAIGGSG	Regulates β1 binding to p27^Kip1^?	?	?	Outer surface of the β-ring, close to β1 from the other β-ring	Regulates CP integrity	No	Yuan et al., 2013

^*^ Estimated positions of sites that are not visible in available 26S proteasome structures

^#^ To be published (personal communication with A. L. Goldberg)

Note: A total of 11 phosphosites that have been studied using low-throughput methods (i.e. “LTP” ≥ 1 in PhosphoSitePlus) are summarized here. The roles they play in proteasome function and how they are regulated are listed. Their exact or predicted positions on the 26S proteasome complex can be seen in Fig. 1

### CK2

Similar to PKA, protein kinase CK2 has a ubiquitous expression profile among tissues and cell types, and is one of the first kinases reported to co-purify with the proteasome from mammalian sources and phosphorylate distinct α subunits (Ludemann et al., 1993; Castano et al., 1996; Mason et al., 1996). Interestingly, CK2 orthologs were shown to phosphorylate 20S subunits of fungi and plants (Umeda et al., 1997; Pardo et al., 1998; Murray et al., 2002), suggesting that this may be a conserved and common property of CK2.

In search for a kinase activity that associates with the 20S proteasome from human erythrocytes, Ludemann et al. found that the putative kinase was distinct from PKA (the only known proteasome kinase then) in that it was insensitive to cAMP but could be effectively blocked by heparin, an inhibitor of CK2. Western blot analysis confirmed the presence of CK2 in 20S proteasome preparations, and *in vitro* phosphorylation of a specific 30 kDa subunit could be eliminated by immunodepletion of CK2 from the proteasome sample. However, CK2 phosphorylation did not seem to affect 20S proteasome activity (Ludemann et al., 1993). A few years later, two groups independently reported that two α subunits, α7/PSMA3/C8 and α3/PSMA4/C9, were predominantly phosphorylated in cells labeled with ^32^P-orthophosphate, and the phosphosites were mapped to serine residues (Castano et al., 1996; Mason et al., 1996). Indeed, α7 has an acidic C-terminal tail that contains two conserved serines (ES_243_LKEEDES_250_DDDNM), both of which could be phosphorylated by CK2 (recognizing S/TXXE/D/pS motifs) *in vitro* as shown by mutagenesis studies (Castano et al., 1996; Bose et al., 2004). Nonetheless, none of these studies proved that CK2 is the physiological kinase for 20S phosphorylation.

What is the functional relevance of such phosphorylations? Rivett’s group demonstrated that α7 and α3 were phosphorylated in both 20S and 26S proteasomes (Mason et al., 1996), but they were much dephosphorylated by interferon gamma (IFN-γ) treatment with a concomitant decrease of 26S proteasome content and increase in 11S/PA28-containing proteasomes in cells (Bose et al., 2001). α7 mutants with either S243 or S250 or both sites changed to alanine seemed to be excluded from 26S complexes. Therefore, α7 appears to be constitutively phosphorylated to stabilize the 26S proteasome. Its dephosphorylation following IFN-γ signaling promotes the switch to 11S-activated proteasomes that are important for downstream immune responses (Rivett et al., 2001; Bose et al., 2004). However, whether IFN-γ specifically induces α7 dephosphorylation at S243/S250 and the underlying mechanism have not been elucidated.

It is noteworthy that α7-S250 is by far the most frequently detected proteasome phosphorylation site (found in >900 spectra curated at PhosphoSitePlus). Treating cells with phosphatase inhibitors Calyculin A and okadaic acid did not further increase S250 phosphorylation (Mason et al., 1998), indicating that it is probably constitutive as revealed by a recent MS study (Gersch et al., 2015). Interestingly, throughout evolution, the very C-terminal end of α7 is rich in acidic residues. In *S. cerevisiae* it also contains three phosphorylation sites that are important for binding to the proteasome quality control factor, Ecm29 (Wani et al., 2016). Whether this holds true for human α7 is unknown. In addition, S250 phosphorylation changes during stem cell differentiation, cell cycle and with exposure to anti-cancer drugs (Brill et al., 2009; Dulla et al., 2010; Schmidt et al., 2011; Imami et al., 2012). It is unclear how this happens since CK2 is generally considered to be always active. In fact, there is even evidence against CK2 since its inhibition actually increased α7-S250 phosphorylation (Franchin et al., 2015). Therefore, the regulation and biological function of this heavily phosphorylated site remain to be rigorously examined (Table 1).

### CaMKIIα

Neuronal synapse formation, maintenance, and plasticity involve drastic changes in the composition of synaptic proteins, and the UPS plays an essential role in controlling local protein turnover during these processes (Ehlers, 2003; Bingol and Sheng, 2011). In response to neuronal activity, the 26S proteasome complex not only becomes physically sequestered in dendritic spines (Bingol and Schuman, 2006) but also exhibits elevated activity (Djakovic et al., 2009). Intriguingly, both phenomena depend on the kinase CaMKIIα, one of the most abundant proteins in the brain and a master regulator of synapses.

Patrick and colleagues first reported that treating hippocampal neurons with bicuculline (BIC, to upregulate action potentials) increased proteasome activity while tetrodotoxin (TTX, an action potential blocker) produced the opposite effect. BIC-induced proteasome activation in neurons was abrogated by CaMKII inhibitors, whereas the constitutively active T286D mutant (mimicking the autophosphorylated form) of CaMKIIα was sufficient to increase proteasome activity in both neurons and 293T cells. Purified CaMKIIα phosphorylated the aforementioned Rpt6-S120 site *in vitro* and was therefore established as a new proteasome-regulating kinase (Djakovic et al., 2009).

Soon after, CaMKIIα was demonstrated to be a PIP *in vivo* as it co-purifies with 26S proteasomes from synaptosome-enriched fractions of rat forebrain. Autophosphorylated CaMKIIα shows better proteasome binding and is both necessary and sufficient for proteasome redistribution to dendritic spines upon NMDA stimulation, resulting in efficient synaptic protein degradation (Bingol et al., 2010). Although this scaffolding function was shown to be independent of CaMKIIα kinase activity toward Rpt6-S120, proteasomes containing the Rpt6-S120D mutant seemed to be more resistant to detergent extraction in hippocampal neurons (Djakovic et al., 2012). Therefore, CaMKIIα-mediated Rpt6-S120 phosphorylation may also contribute to proteasome tethering at the spines. Functionally, blockade of this phosphorylation by the S120A mutation or CaMKIIα inhibition reduces synaptic activity and prevents activity-induced spine growth (Djakovic et al., 2012; Hamilton et al., 2012). Furthermore, in rats, fear conditioning as well as fear memory retrieval increases proteasome activity and Rpt6-S120 phosphorylation in amygdala in a CaMKIIα-dependent manner. Both pharmacological inhibition of CaMKIIα and S120A knock-in lead to defects of learning and memory in rodents (Jarome et al., 2013; Jarome et al., 2016 and personal communications with G. Patrick), strongly supporting the physiological significance of such phospho-regulation of the proteasome (Table 1).

### DYRK2

The cell cycle is driven by ordered and finely regulated proteasomal degradation of many proteins such as cyclins, cyclin-dependent kinase (CDK) inhibitors, and licensing and check point factors. As a central regulator of cell cycle, the 26S proteasome itself contains multiple residues that are phosphorylated at different cell cycle stages (Dephoure et al., 2008; Nagano et al., 2009; Olsen et al., 2010; Kettenbach et al., 2011). The first kinase that has a clear role in cell cycle-dependent proteasome phosphorylation turns out to be DYRK2 (Guo et al., 2016), a member of the dual-specificity tyrosine phosphorylation regulated kinase family (Becker, 2012).

Unlike PKA, CK2, and CaMKIIα that were all identified with targeted approaches, DYRK2 was discovered in an unbiased screen for kinases that phosphorylate a particular site of the proteasome, Rpt3-Thr25 (Table 1). This phosphosite was first detected in mitotic HeLa cells by MS (Dephoure et al., 2008) then confirmed in multiple cell types with a phospho-specific antibody (Guo et al., 2016). As seen with Rpt6-S120 and Rpn6-S14, phosphorylation of Rpt3-T25 also upregulates proteasome activity towards peptide and folded protein substrates *in vitro* and in cells. Interestingly, Rpt3-T25 phosphorylation occurs only in actively proliferating cells, with its level being low in G_1_ phase but markedly elevated during S, G_2_, and M phases of the cell cycle. CRISPR/Cas9-mediated knock-in of Rpt3-T25A mutation, which reduces endogenous proteasome activity, causes marked accumulation of cell cycle inhibitors such as p21^Cip1^ and p27^Kip1^ during S-to-G_2_/M transition and impedes cell proliferation. This is the first example that a phosphorylation event coordinates proteasome activity with cell cycle progression (Guo and Dixon, 2016; Guo et al., 2016).

Given the biochemical and biological importance of Rpt3-T25 phosphorylation, a human kinase cDNA library from the laboratory of the late Dr. Susan L. Lindquist (Taipale et al., 2012) was used to screen for the responsible kinase(s). An unexpected kinase, DYRK2, was found to strongly phosphorylate T25 *in vitro* and in cells, while its disruption by CRISPR/Cas9 essentially abolished the phosphorylation. The adjacent sequence of T25 (RPXT) is conserved in vertebrates and is a known substrate motif of DYRK family kinases (Howard et al., 2014). DYRK2-null cells exhibit lowered proteasome activity and slowed proliferation, phenocopying Rpt3-T25A knock-in. In keeping with the dynamics of Rpt3-T25 phosphorylation during cell cycle, DYRK2 itself is transcriptionally induced upon S phase entry. This leads to upregulation of T25 phosphorylation and efficient proteasomal degradation of key proteins like p21^Cip1^ and p27^Kip1^, which in turn facilitates cell cycle progression (Guo et al., 2016; Huibregtse and Matouschek, 2016).

As discussed above, proteasome activity can be manipulated *in vivo* by changing its phosphorylation status, offering new possibilities for proteasome-oriented therapies. Several types of cancer are exquisitely dependent on proteasome activity for survival, including basal-like triple negative (ER^−^/PR^−^/HER2^−^) breast cancer (Petrocca et al., 2013). Inactivation of DYRK2 sensitized these cancer cells to the proteasome inhibitor Bortezomib *in vitro*, and blockade of Rpt3-T25 phosphorylation significantly attenuated their tumorigenecity *in vivo* (Guo et al., 2016). In addition, *DYRK2* gene is amplified in a considerable fraction of cancers (Santarius et al., 2010) and its mRNA level negatively correlates with clinical outcome of breast cancer patients (Guo et al., 2016). Therefore, simultaneous targeting of DYRK2 and the proteasome may be a promising combinatorial approach for treating certain cancers, as supported by preliminary data from ongoing research.

### PKG

The post-mitotic cardiomyocytes, like neurons, are particularly vulnerable to damaged, misfolded, and aggregated proteins. With a major role in the clearance of cytotoxic proteins, the UPS is essential for the health of cardiomyocytes but its function is often impaired in heart disease. Enhancing the degradative capacity of cardiac proteasomes may therefore provide a way for heart disease control and treatment. An attractive strategy appears to be through activation of protein kinase G (PKG), a key regulator of cellular functions in the cardiovascular system (Rainer and Kass, 2016).

PKG is activated by the small-molecule second messenger cyclic guanosine 3’-5’ monophosphate (cGMP). Sildenafil, a phosphodiesterase 5 inhibitor which raises cGMP levels, enhanced proteasome activity in neonatal rat ventricular myocytes, while PKG inactivation caused the opposite effects (Ranek et al., 2013). Importantly, *in vivo* administration of sildenafil effectively reduced protein aggregation and hypertrophy in cardiac tissues of transgenic mice expressing CryAB^R120G^, a mutant protein whose misfolding causes desmin-related cardiomyopathy. Indirect evidence suggested that PKG may be involved Rpt6 and β5 phosphorylation in cells (Ranek et al., 2013). Although the phosphorylation sites remain to be elucidated, this study provides mechanistic insights into the established anti-hypertrophy function of sildenafil and therefore may have profound clinical importance (Gillette and Hill, 2013).

### Other Ser/Thr kinases

Multiple lines of evidence suggest that polo-like kinase 1 (Plk1) is a proteasome kinase. Plks are activated during G_2_/M transition of the cell cycle and regulate key events of mitosis (van de Weerdt and Medema, 2006). Plk1, probably via its polo-box domain, can directly interact with most 20S subunits and some 19S subunits (Lowery et al., 2007; Dephoure et al., 2008). The 20S proteasome has been shown to be phosphorylated (especially at the α3 and α7 subunits) and activated *in vitro* by Plk1 pre-treated with OA or mitotic lysates (Feng et al., 2001). On the other hand, Plk1 inhibitors reduce or block the phosphorylation of several proteasome subunits (Grosstessner-Hain et al., 2011; Kettenbach et al., 2011; Santamaria et al., 2011). The exact phosphorylation sites and the function of Plk1-mediated proteasome regulation in mitosis remain to be determined.

As opposed to the above examples, several other Ser/Thr kinases appear to negatively regulate proteasome activity. For example, the p38 MAPK phosphorylates Rpn2-Thr273 upon sorbitol-induced osmotic stress, leading to decreased peptidase activity of the proteasome without changing its assembly (Lee et al., 2010b, Table 1). This phosphorylation also increases with high NaCl treatment (Wang et al., 2014). Another MAPK, ERK2, could phosphorylate Rpn2-T273 *in vitro* (Tsai et al., 2015). In brain slices from mouse hippocampal CA1 region, long-term potentiation (LTP) stimulation decreased Rpn2-T273 phosphorylation by an unknown mechanism (Li et al., 2016), presumably leading to an increase in proteasome activity as seen with CaMKIIα activation.

Other stress inducers, such as H_2_O_2_ or the DNA topoisomerase inhibitor etoposide that signal through the ASK1-JNK1 pathway, can also inhibit the proteasome. This inhibition requires ASK1, which can bind the ATPase subunits and phosphorylate Rpt5, leading to decreased proteasome ATPase activity hence substrate degradation *in vitro* and *in vivo* (Um et al., 2010). Another possible inhibitory kinase is AMPK. Several AMPK activators, including AICAR, Metformin and A-769662, could downregulate 26S proteasome activity, while genetic deletion of AMPKα2 did the opposite (Moreno et al., 2008; Viana et al., 2008; Wang et al., 2010). However, the mechanisms and physiological meanings of these regulations have yet to be uncovered.

### Abl/Arg and proteasome tyrosine phosphorylation

C-Abl and the related protein Arg (Abl-related gene product) are multi-functional tyrosine kinases and are so far the only tyrosine kinases shown to directly phosphorylate and regulate the proteasome (Liu et al., 2006; Li et al., 2015). Abl was found in a yeast two-hybrid screen to interact with the 20S subunit α4/PSMA7. Both Abl and Arg phosphorylate α4 at two conserved tyrosine residues, Y106 and Y153 (Table 1). However, these phosphorylations seem to have opposite effects on proteasome function. Y153 phosphorylation reduced proteasome activity *in vitro* and in cells, whereas Y106 phosphorylation protected α4 from degradation by the proteasome. As a result, the 26S holoenzyme was low in abundance but high in activity in *Abl*/*Arg* double knockout cells, leading to little net change in the overall degradation capacity as compared to WT cells. Albeit the proteotoxic effect of oxidative stress appeared more evident in the absence of Abl and Arg (Li et al., 2015), the physiological significance of α4 tyrosine phosphorylation remains nebulous.

Except for these studies, almost nothing is known about the function and regulation of proteasome tyrosine phosphorylation. Many tyrosine residues of human 26S proteasome reported to be phosphorylated are conserved even in yeast, which is surprising in light of the limited overlap of proteasome phosphosites between the two species and the evolutionary reduction of tyrosine phosphorylation in higher organisms (Tan et al., 2009). Since yeast genome does not encode conventional tyrosine kinases (Manning et al., 2002) and yeast proteasome is rarely tyrosine-phosphorylated (PhosphoGrid), those conserved tyrosine residues presumably play structural rather than regulatory roles. As mentioned earlier, the HbYX motif at the C-terminal tails of Rpt2, 3, and 5 are critical for bolting the 19S and 20S particles together. However, the penultimate tyrosines within this motif of human Rpt2 and Rpt3 seem to be more frequently phosphorylated than any other pY site of the 19S RP. Such phosphorylations would undoubtedly preclude RP-CP interaction, therefore must happen on free 19S RP (if they truly happen in cells), perhaps as a prerequisite or checkpoint for 19S RP assembly and/or 26S proteasome formation.

MS results indicate that pY sites are spread all over the 26S complex and they constitute an astounding 1/3 (150/455) of all known proteasome phosphosites. Moreover, 11 out of the top 12 most frequently detected proteasome phosphorylations (i.e. sites with HTP > 100 from PhosphoSitePlus) occur on tyrosine residues (Table S1). Such over-representation of pY in the proteasome complex is, at first sight, quite striking as pY is generally perceived as a very minor portion of the whole phosphoproteome (Hunter and Sefton, 1980; Olsen et al., 2006; Sharma et al., 2014; Bian et al., 2016). However, it should be emphasized that most proteasome tyrosine phosphorylations were observed by MS following treating cells with pervanadate (a potent non-specific inhibitor of protein tyrosine phosphatases, or PTPs) and enrichment of peptides with anti-pY antibodies. In fact, few tyrosines have been found phosphorylated on endogenous proteasome proteins from mouse tissues (PhosphoMouse), and affinity-purified proteasomes from untreated human cells contain very little pY signal as shown by Western blot (our unpublished results). Together, these observations strongly suggest that, although the proteasome may be constantly phosphorylated by tyrosine kinases, the modification must occur at a low stoichiometry and are very sensitive to dephosphorylation by PTPs. Identification of the relevant tyrosine kinases and PTPs will shed light on why proteasome tyrosine phosphorylation happens but is kept under such tight control.

On the other hand, numerous studies have reported significant increases of proteasome tyrosine phosphorylation, which invariably took place in cancer cells with aberrant tyrosine kinase signaling (Rush et al., 2005; Gu et al., 2006; Rikova et al., 2007; Guo et al., 2008; Luo et al., 2008; Iliuk et al., 2010; Bai et al., 2012; Drake et al., 2012; Johnson et al., 2012). Most of the deregulated pY sites locate on 20S subunits, especially α2, whose Y24, Y57, Y76, and Y101 residues appear to be “hot spots” targeted by oncogenic tyrosine kinases. Considering the prevalent upregulation of proteasome activity in various types of cancer (Hoeller and Dikic, 2009), in-depth understanding of proteasome tyrosine phosphorylation may provide new insights into cancer pathogenesis, diagnosis, and treatment.

### UBLCP1 and proteasome phosphatases

The generally low stoichiometry of proteasome phosphorylations (especially pY) and the necessity of phosphatase inhibitors for their detection strongly indicate a significant role of dephosphorylation in controlling proteasome functions. However, compared to the kinases, proteasome phosphatases have been even less investigated. Evidence exists that treating the proteasome with common phosphatases (such as PP1 and PP2A family members) or non-specific phosphatases (such as λ-phosphatase, alkaline/acidic phosphatases) can reduce the peptidase activities *in vitro* (Mason et al., 1996; Zong et al., 2006; Zhang et al., 2007a; Kikuchi et al., 2010; Guo et al., 2011). In addition, PP2A subunits and Calcineurin subunits have been shown to be in complex with the 20S proteasome (Zong et al., 2006; Li et al., 2011; Zhang and Wei, 2011), but their roles in proteasome regulation are far from clear. At present, the only physiological proteasome phosphatase that has been functionally characterized is ubiquitin-like domain containing CTD phosphatase 1 (UBLCP1).

UBLCP1 belongs to the haloacid dehalogenase (HAD) family of phospho-Ser/Thr phosphatases that consists of at least seven members in mammals. Unlike founding members of this family, SCP1 and FCP1, which are known to dephosphorylate the C-terminal domain (CTD) of RNA polymerase II (Pol II), UBLCP1 does not interact with or regulate Pol II. Instead, it is targeted to the 26S proteasome via its UBL domain (Guo et al., 2011). In fact, UBLCP1 is the only phosphatase in the human phosphatome that contains a UBL domain, making it the first and only known proteasome-resident phosphatase. Knockdown of UBLCP1 enhances proteasome activity in cells, while *in vitro* UBLCP1 directly dephosphorylates multiple subunits of purified 26S proteasome and reduces its activity. Therefore, UBLCP1 negatively regulates proteasome function, in a manner that relies on both its phosphatase activity and direct interaction with the proteasome. One mechanism for this regulation is that UBLCP1 prevents 19S-20S association (or promotes 26S dissociation), thereby attenuating the overall proteasome activity (Guo et al., 2011). However, the exact phosphosites modulated by UBLCP1 were not identified.

Another important property of UBLCP1 is that it is a nuclear protein without a canonical nuclear localization signal (NLS). Its exclusive nuclear localization is strictly dependent on a single conserved lysine residue (K44) within the UBL domain, which is also critical for UBLCP1 binding to the proteasome. Thus, UBLCP1 selectively downregulates nuclear proteasome without affecting cytoplasmic proteasome (Guo et al., 2011). This work, together with previously mentioned synaptic retention of proteasomes by CaMKIIα (Bingol et al., 2010; Djakovic et al., 2012), demonstrates compartmentalized proteasome regulation and highlights the cellular heterogeneity of proteasomes that is usually masked by using cell/tissue homogenates (Sha et al., 2011; Schmidt and Finley, 2014).

Owing to the unique structure of UBLCP1 catalytic domain (Guo et al., 2011), a small-molecule inhibitor was identified from a high-content screen that is both potent and specific against this phosphatase. Treating cells with this inhibitor caused an increase of nuclear proteasome activity, consistent with UBLCP1 being a negative regulator of nuclear proteasomes (He et al., 2015). This effect is analogous to that of inhibiting USP14, a proteasome-associated deubiquitinating enzyme (Lee et al., 2010a), both employing an “inhibition-of-an-inhibitor” strategy to achieve proteasome activation. Given the impaired proteasome function in neurodegenerative and heart diseases, these compounds may represent a different approach than PKA activators (See above) for therapeutics.

## HOW DOES PHOSPHORYLATION MODULATE PROTEASOME FUNCTION?

In principle, a phosphorylation event can positively or negatively impact any aspect of proteasome function and any step during its biogenesis. This view has been more or less proven by available examples from largely isolated studies as described above. Collectively, phosphorylation can regulate (i) protein stability and abundance of certain subunits (Li et al., 2015), (ii) proteasome assembly, stability or composition (Satoh et al., 2000; Bose et al., 2004; Guo et al., 2011; Lokireddy et al., 2015), (iii) subcellular localization of the proteasome (Benedict and Clawson, 1996; Bingol et al., 2010; Djakovic et al., 2012), (iv) PIP binding (Wani et al., 2016), (v) substrate recognition (Satoh et al., 1995; Yuan et al., 2013), and (vi) enzymatic activities (Mason et al., 1996; Liu et al., 2006; Zong et al., 2006; Djakovic et al., 2009; Bingol et al., 2010; Guo et al., 2016).

Phosphorylations often take place at flexible loops and disordered regions of proteins (Holt et al., 2009), posing a potential challenge for structural analysis. Indeed, despite biochemical and functional evidence, no structural basis is available for any of the proteasome phosphosites, leaving a big gap in our understanding of how exactly their phosphorylations alter proteasome properties. Take DYRK2-mediated Rpt3-T25 phosphorylation as an example, it is located near the extreme N-terminus of Rpt3, a highly dynamic region invisible in all crystal and cryo-EM structures of the proteasome. However, it is likely tucked under Rpn2 and regulates the rotation of Rpn2 and 19S lid around the fulcrum formed by the Rpt3-Rpt6 coiled-coil (Matyskiela et al., 2013). Such rotation is believed to be coupled with substrate unfolding and translocation, consistent with biochemical studies showing that Rpt3-T25 phosphorylation enhances substrate-stimulated ATPase activity of the proteasome (Guo et al., 2016). Interestingly, although T25 is not conserved in yeast Rpt3, the latter has a nearby residue, Thr8, that has been found to be dynamically phosphorylated during cell cycle (L. Huang, personal communication). Despite that the exact site and (probably) the responsible kinase are different, it is tempting to postulate that phosphorylation at the very N-terminus of human and yeast Rpt3 may have similar functions, therefore might be considered as “functionally conserved”. Such scenario has been observed for other phosphorylation events (Holt et al., 2009) as well as other post-translational modifications (Xu et al., 2013). Figure 1 illustrates the observed or predicted positions of several functionally relevant phosphosites on the high-resolution cryo-EM structure of human 26S proteasome (Huang et al., 2016), and their speculated roles in proteasome regulation are summarized in Table 1. Clearly more biophysical and structural insights are needed to fully explain the molecular mechanisms for phosphoregulation of the proteasome.

## PROSPECTS

How do we go further in understanding proteasome phosphorylation? The simple answer is that we need to go broader and go deeper. On one hand, previous research on proteasome phosphorylation has been isolated and mostly relied candidate approaches. More systematic screens are needed to identify kinases and phosphatases that regulate proteasome activity and phosphorylation profile. Libraries of cDNAs, shRNAs, sgRNAs, and small-molecule inhititors are readily available for this purpose and have been successfully used (Chou and Deshaies, 2011; Guo et al., 2016). Knowledge about proteasome kinases and phosphatases is instrumental because it not only allows for direct manipulation of proteasome phosphorylation for biochemical analysis *in vitro* and *in vivo*, but also helps to elucidate the biological meaning of proteasome phosphorylation by connecting it with signaling pathways and cellular activities.

On the other hand, description of proteasome phosphorylation in proteomic studies has been superficial and it is necessary to dig deeper into the function and regulation of individual events. Phospho-specific antibodies, site-directed mutagenesis and targeted MS have been and will still be the prevalent methods for characterizing the phosphosites. A high quality phospho-antibody would be particularly useful for determining the intracellular localization of phosphorylated proteasomes, a critical question with little investigation. It will also facilitate the search for relevant kinases and phosphatases. Overexpression of proteasome subunits bearing phosphosite mutations may or may not yield a change in proteasome activity in cells, since the endogenous wild-type proteins are usually highly abundant, and epitope-tagged exogenous mutants may not be fully incorporated into the proteasome complex. In this sense, homozygous knock-in of point mutations using gene editing tools such as CRISPR/Cas9 can unequivocally reveal the functional requirement of proteasome phosphorylation, as has been demonstrated (Guo et al., 2016). New methods and instrumentation for quantitative MS are needed for more sensitive and accurate capture of transient, dynamic, and low-abundance phosphorylations.

On a separate note, in addition to serine, threonine and tyrosine, phosphorylation also occurs on other residues such as histidine. The recent development of monoclonal antibodies against phospho-histidine has redefined our view of such modifications in human cells (Fuhs et al., 2015). Intriguingly, a considerable fraction of the histidine-phosphorylated proteome is comprised of proteasome subunits (Fuhs et al., 2015). This finding recalls previously identified phosphohistidine residues in 20S subunits (Yano et al., 1999), and yet the physiological function and regulation of proteasome histidine phosphorylation are not known.

## CONCLUDING REMARKS

All of the above research has vividly demonstrated the heterogeneity and complexity of the proteasome, and has shattered the stereotypic view that the proteasome is a “boring” house-keeping machinery. Reversible phosphorylation fine-tunes proteasome activity and adds a new layer of regulation to proteostasis, the basis of all cellular life. The exquisite control of conserved phosphosites reflects highly specialized needs for coordinating proteasome function with specific physiological activities during evolution, which we still know very little about. Research in this field urgently calls for advanced experimental systems and tools such as single-molecule recording, *in situ* electron cryotomography, super-resolution microscopy, quantitative cross-linking MS, as well as other new biochemical/biophysical methods, pharmacological agents, antibodies, and animal models (Pack et al., 2014; Asano et al., 2015; Lu et al., 2015). Development of these tools will also benefit research on other aspects of proteasome regulation and on macromolecular complexes in general.

Proteasome phosphorylation is not only of biological significance but also clinically relevant. Proteasome inhibitors as anti-cancer drugs cannot distinguish between cancer cells and normal cells, which, however, often differ drastically in their phospho-signaling. Therefore, targeting both the proteasome itself and its modulators deregulated in cancer cells is expected to increase the efficacy of proteasome inhibitors, improve drug selectivity, and even partly overcome drug resistance. Conversely, proteasome activation can be beneficial in treating neurodegenerative and heart diseases, which can be achieved by kinase activators (as in the case of PKA and PKG) or phosphatase inhibitors (as in the case of UBLCP1). In-depth understanding of proteasome phosphorylation will greatly expand the repertoire of biochemicals that can be used for proteasome modulation, providing more choices for proteasome-based regimens in the clinic.

## Electronic supplementary material

Below is the link to the electronic supplementary material.

Supplementary material 1 (PDF 2442 kb)

Supplementary material 2 (XLSX 55 kb)
