# Supplementary material for: Reversible phosphorylation of the 26S proteasome
Source: Protein Cell. 2017 Mar 3;8(4):255–72. doi: 10.1007/s13238-017-0382-x (PMC5359188; doi:10.1007/s13238-017-0382-x)

Supplementary information

Supplementary Table 1. A complete list of all 455 phosphosites of human 26S proteasome.

The number of detections of each site is indicated by LTP (based on low-throughput methods with site-specific information) and HTP (based on high-throughput methods). See www.phosphosite.org for detailed definition.

Supplementary Figure 1. Expanded SeqLogos of human proteasome phosphosite motifs.

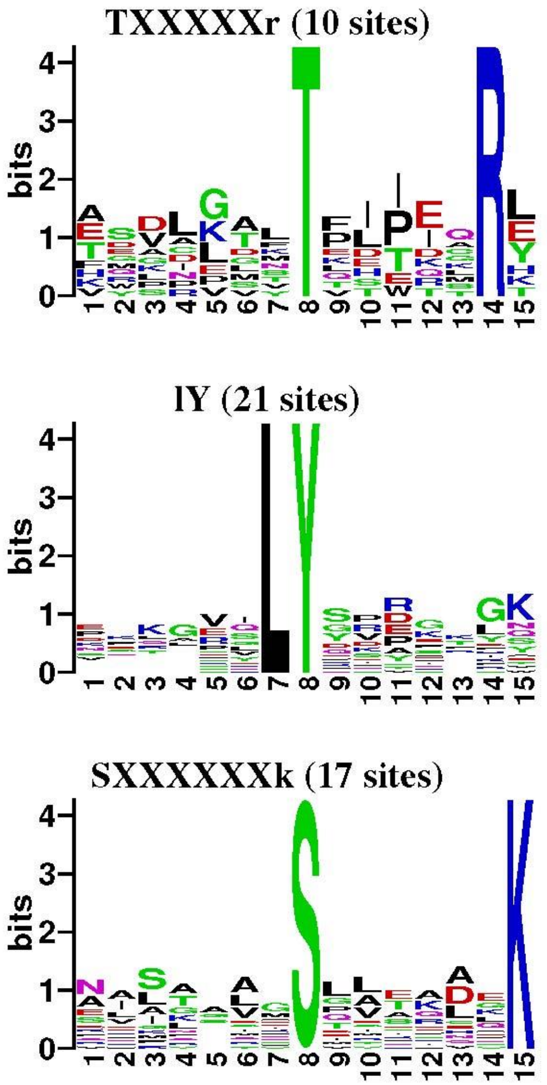

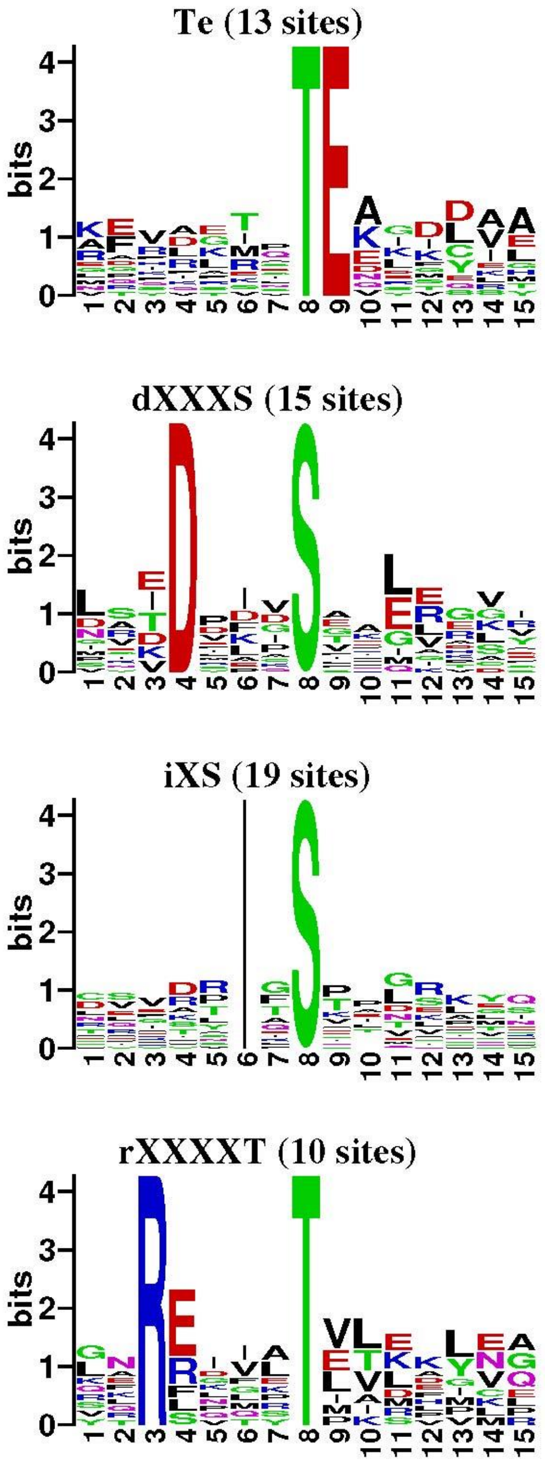
Te (13 sites)
dXXXS (15 sites)
iXS (19 sites)
rXXXXT (10 sites)
bits

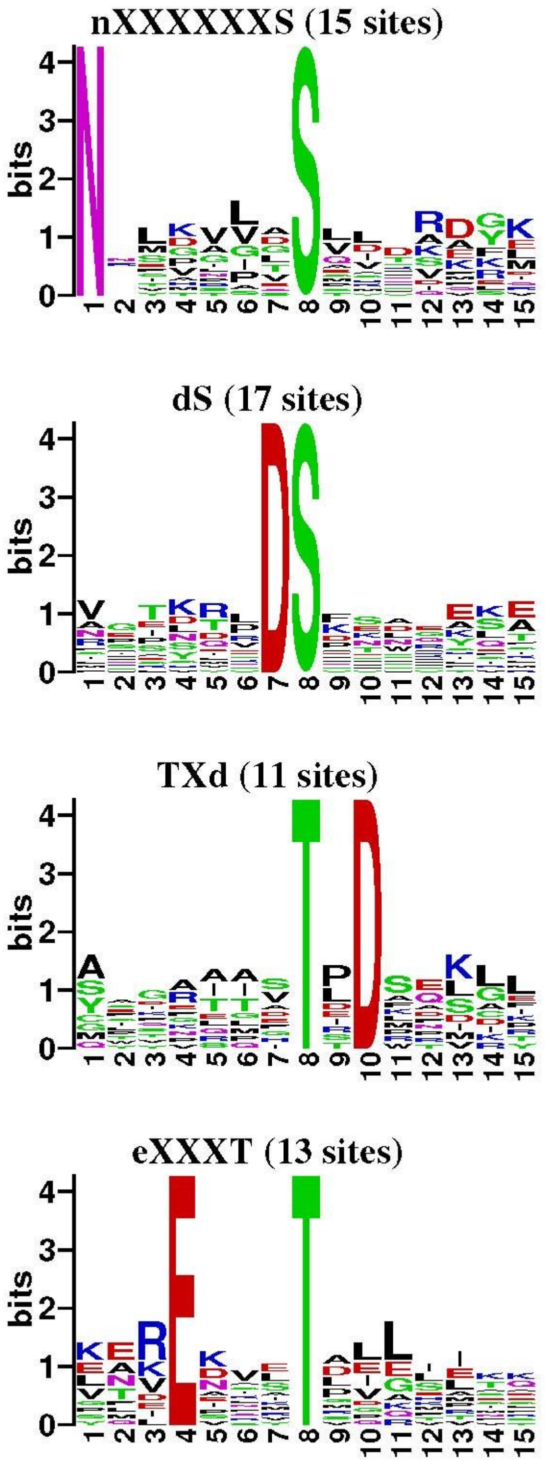
nXXXXXXS (15 sites)
dS (17 sites)
TXd (11 sites)
eXXXT (13 sites)
bits

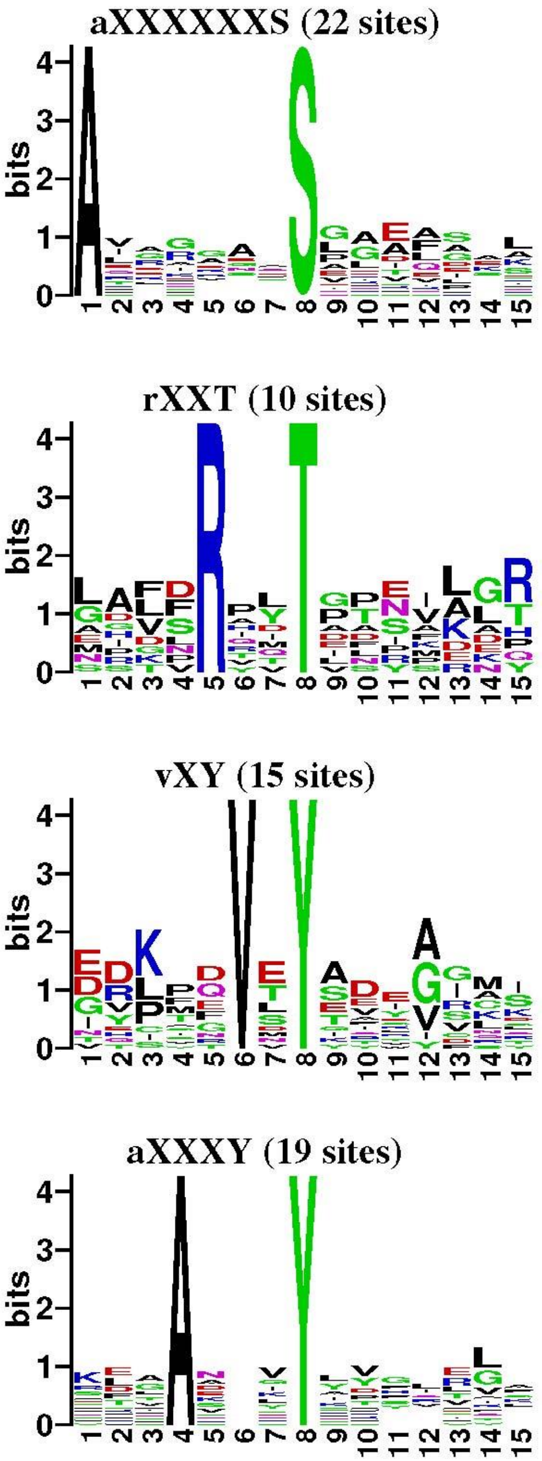

aXXXXXXS (22 sites)
rXXT (10 sites)
vXY (15 sites)
aXXXY (19 sites)
bits

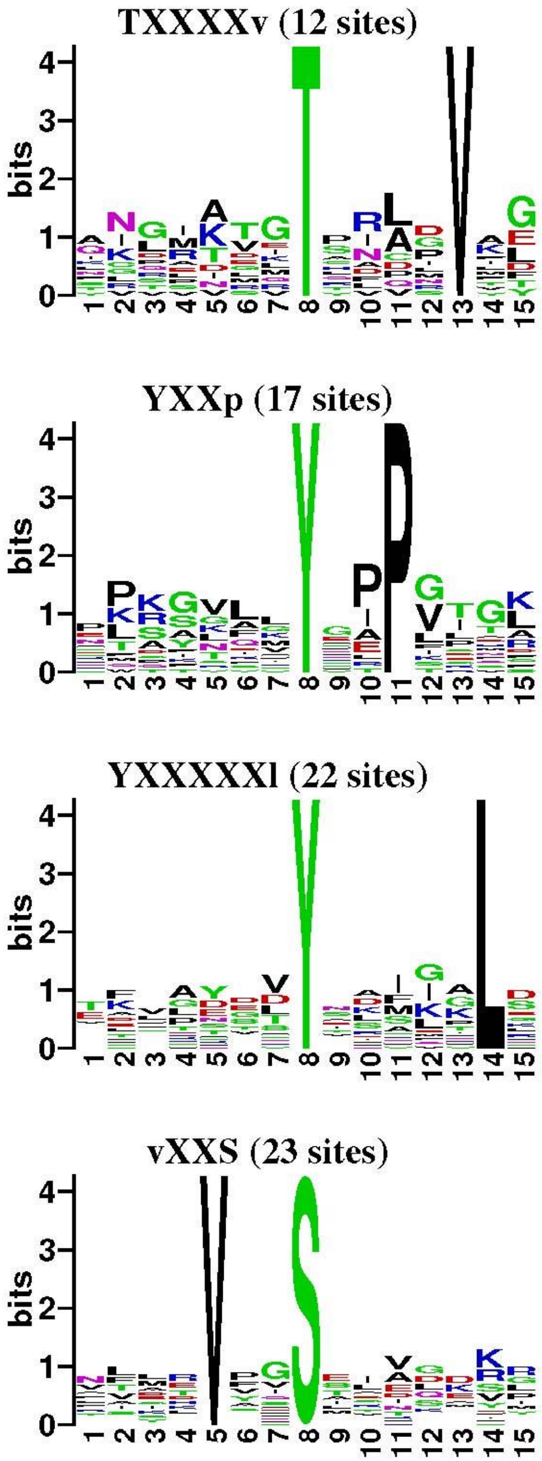
TXXXXv (12 sites)
YXXp (17 sites)
YXXXXXl (22 sites)
vXXS (23 sites)
bits
0
1
2
3
4
1 2 3 4 5 6 7 8 9 10 11 12 13 14 15

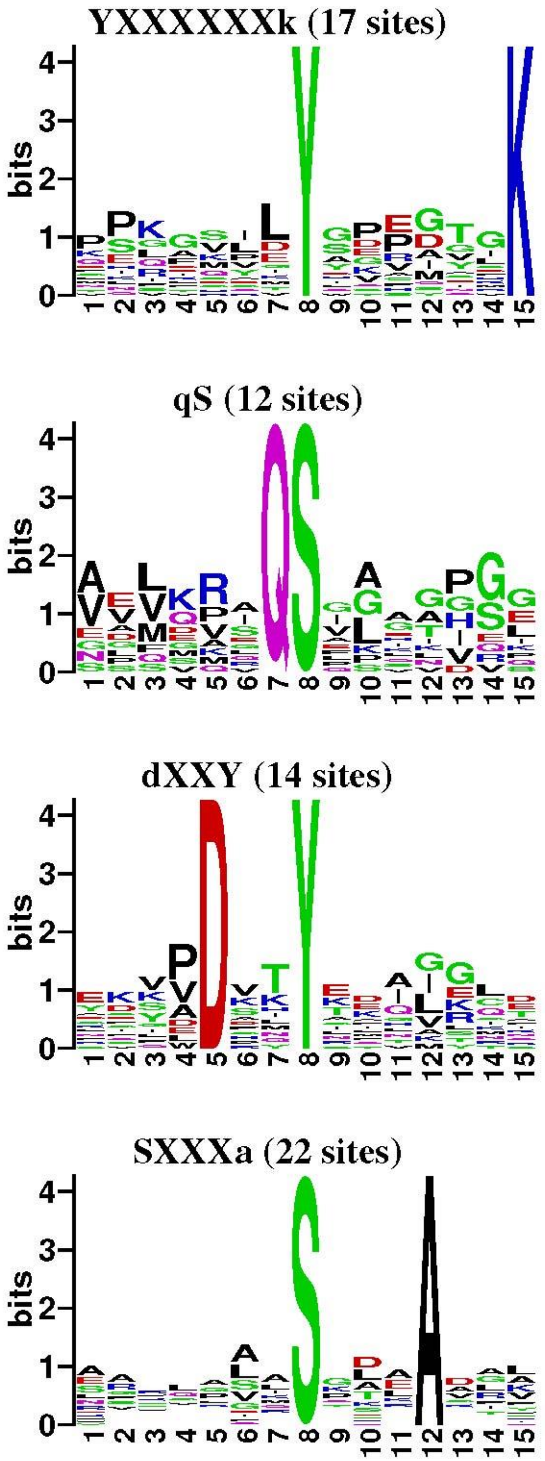
YXXXXXXk (17 sites)
qS (12 sites)
dXXY (14 sites)
SXXXa (22 sites)
bits
0
1
2
3
4
1 2 3 4 5 6 7 8 9 10 11 12 13 14 15

iXXXXS (15 sites)

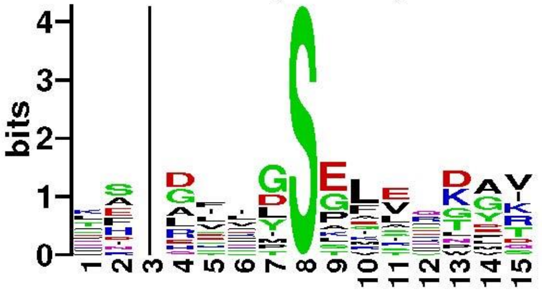

YXXXXXg (17 sites)

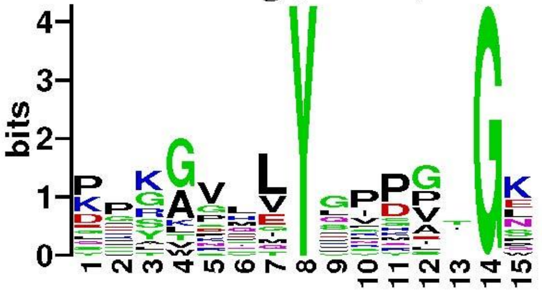

vS (17 sites)

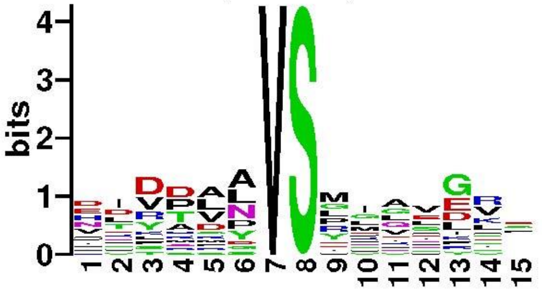

SXXe (28 sites)

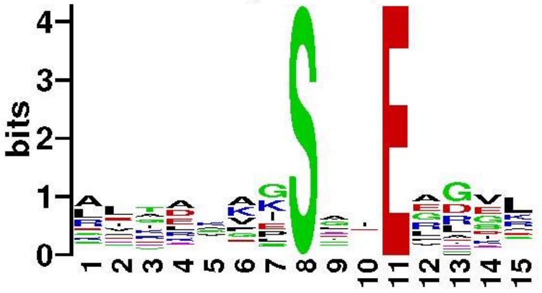

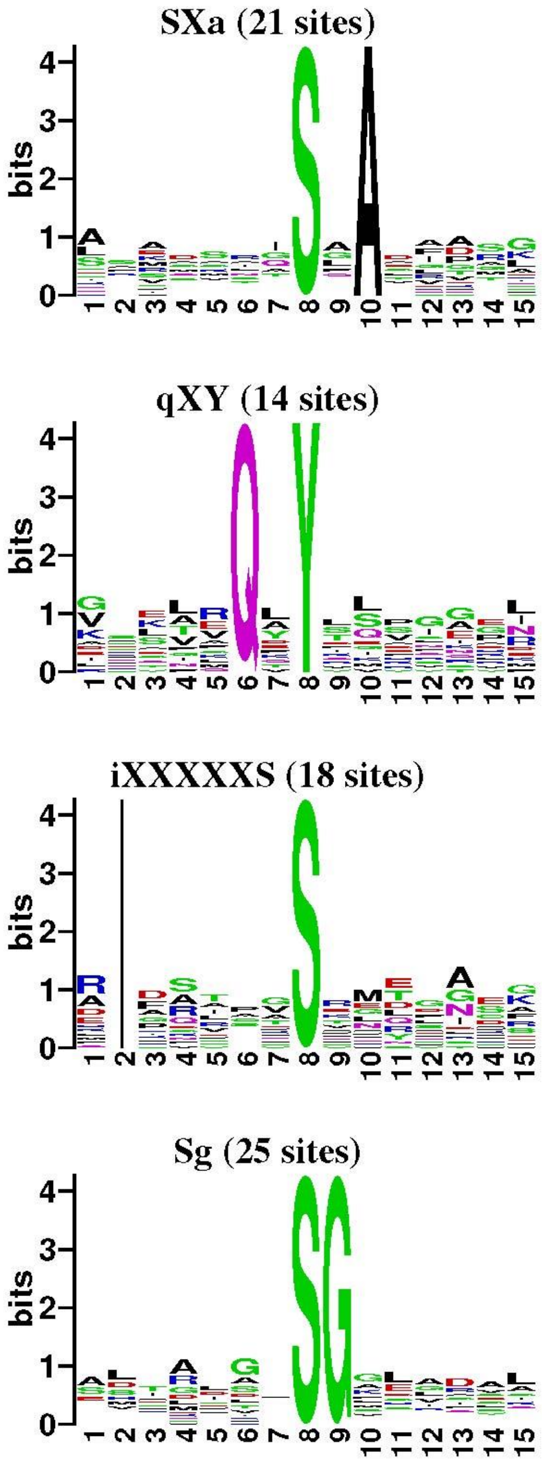

SXa (21 sites)
qXY (14 sites)
iXXXXXS (18 sites)
Sg (25 sites)
bits
0
1
2
3
4
1 2 3 4 5 6 7 8 9 10 11 12 13 14 15

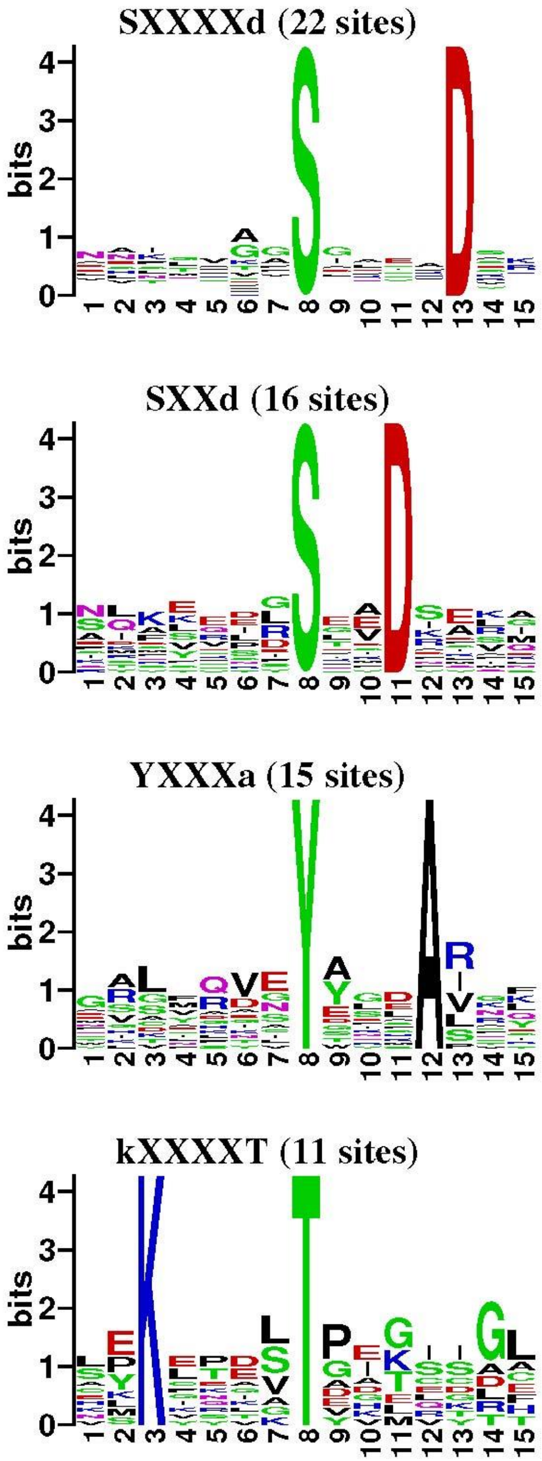
SXXXXd (22 sites)
bits
SXXd (16 sites)
bits
YXXXa (15 sites)
bits
kXXXXT (11 sites)
bits

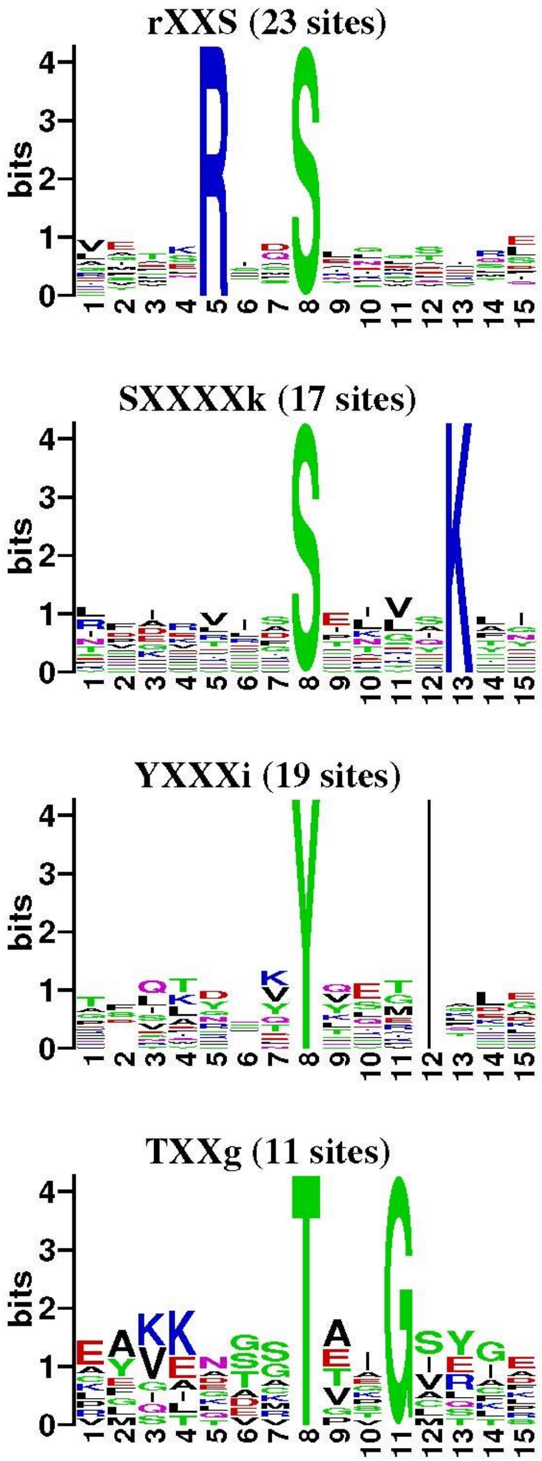
rXXS (23 sites)
SXXXXk (17 sites)
YXXXi (19 sites)
TXXg (11 sites)
bits
0
1
2
3
4
1 2 3 4 5 6 7 8 9 10 11 12 13 14 15

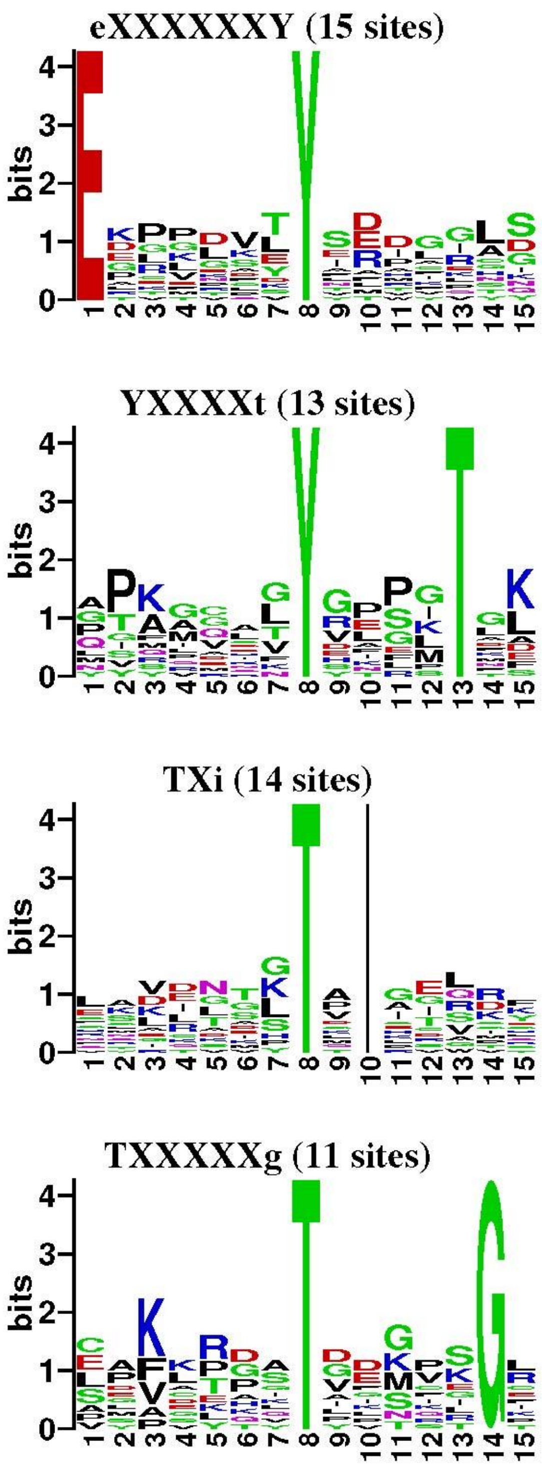
eXXXXXXY (15 sites)
YXXXXt (13 sites)
TXi (14 sites)
TXXXXXg (11 sites)
bits

SXXg (22 sites)

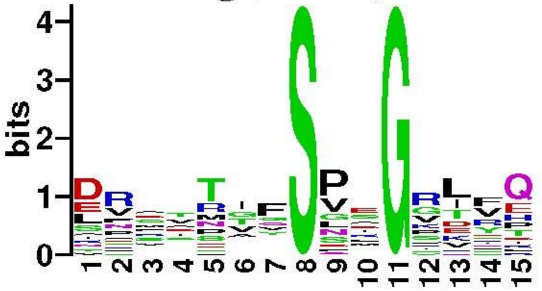

Yy (16 sites)

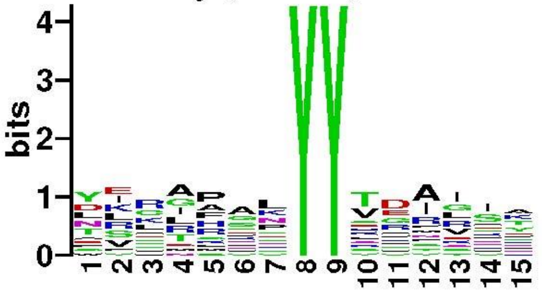

kXXXXXY (15 sites)

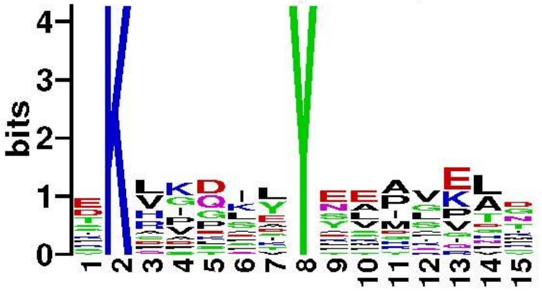

kXXXXXXT (11 sites)

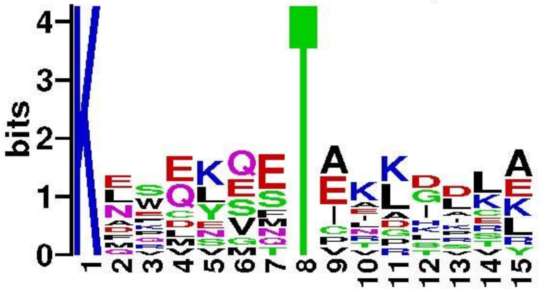

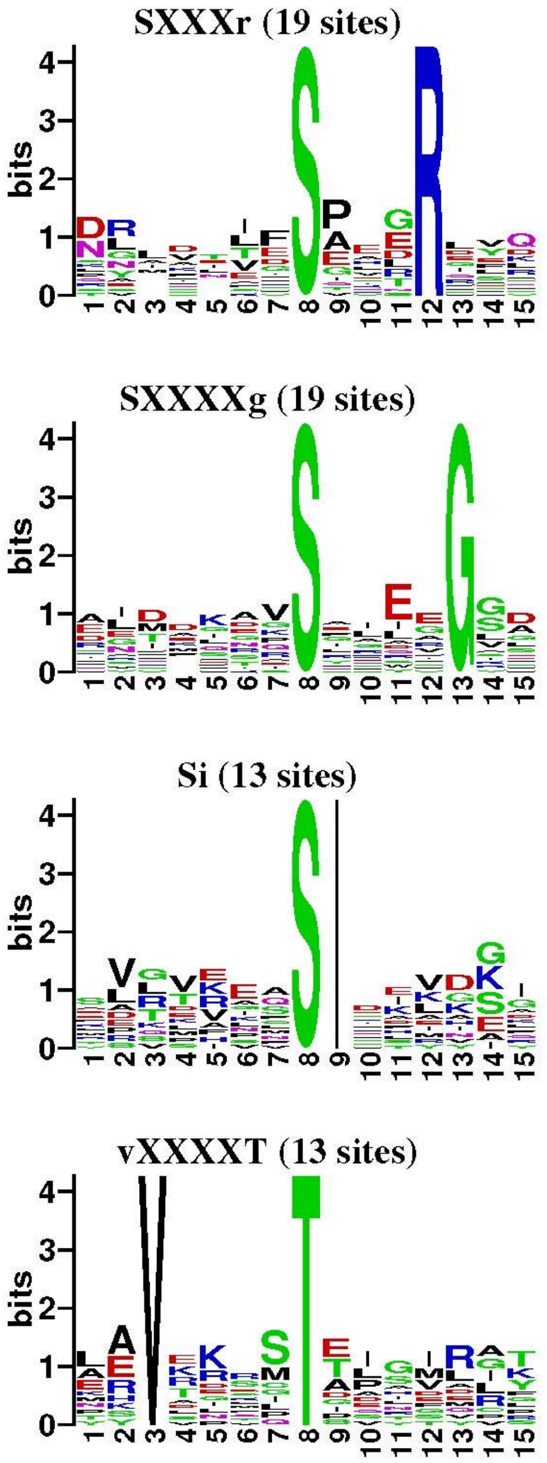
SXXXr (19 sites)
SXXXXg (19 sites)
Si (13 sites)
vXXXXT (13 sites)
bits
0
1
2
3
4
1 2 3 4 5 6 7 8 9 10 11 12 13 14 15

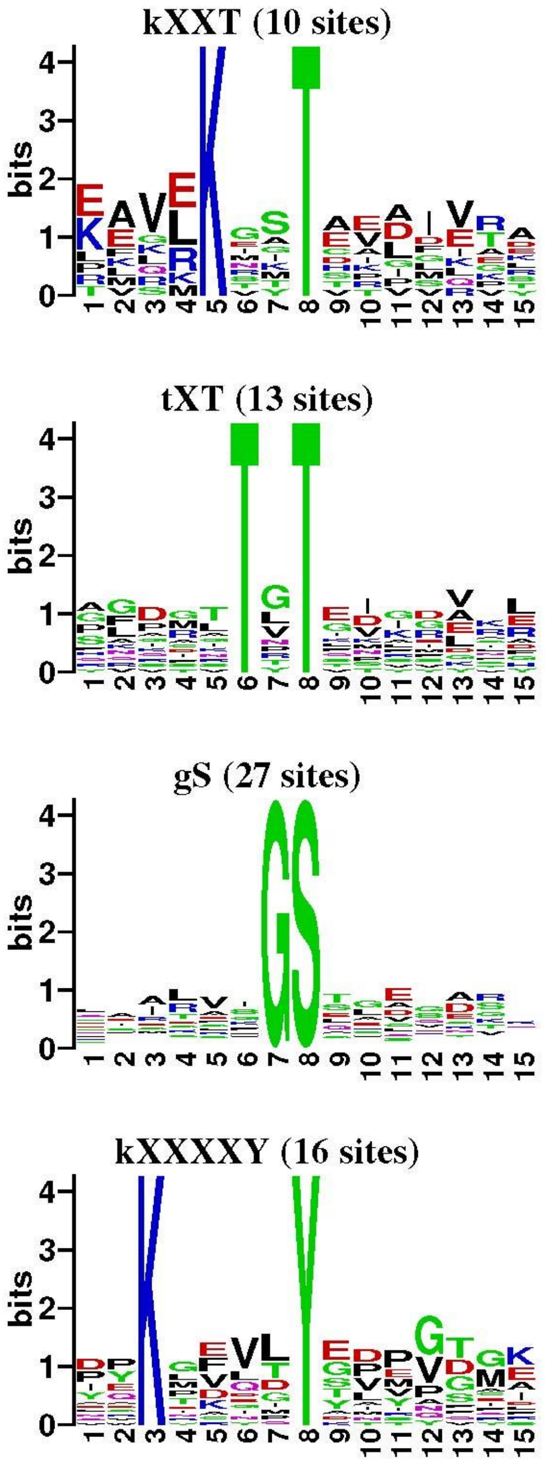
kXXT (10 sites)
bits
tXT (13 sites)
bits
gS (27 sites)
bits
kXXXXY (16 sites)
bits

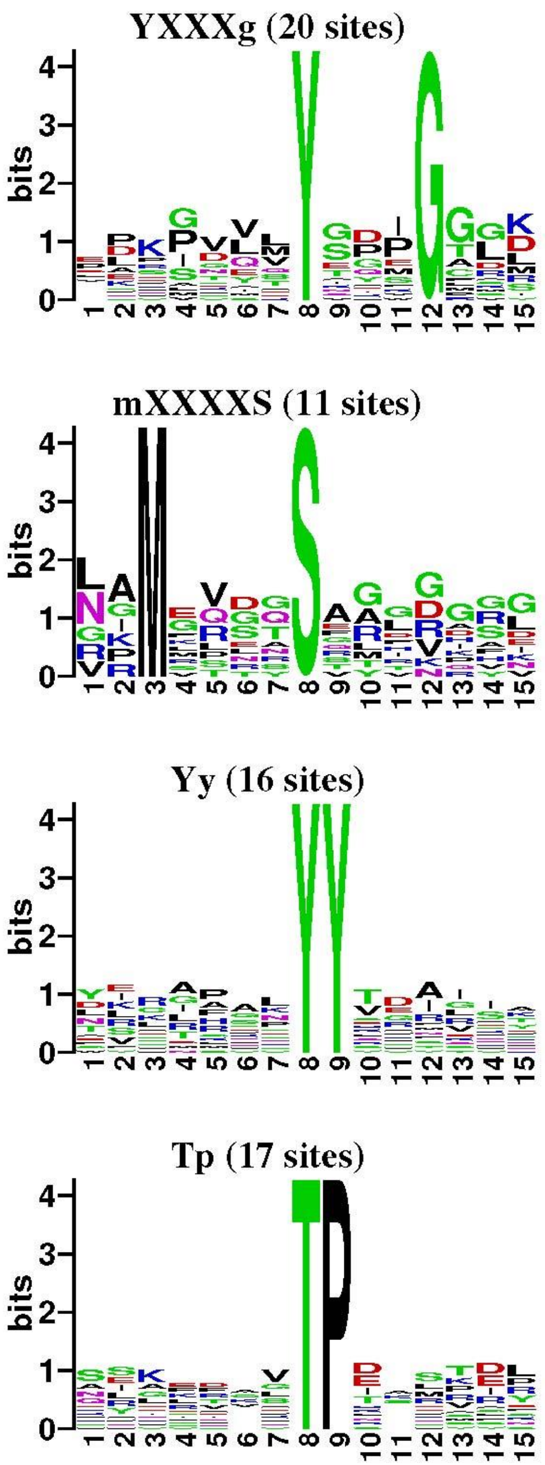
YXXXg (20 sites)
mXXXXS (11 sites)
Yy (16 sites)
Tp (17 sites)
bits

TXXXi (11 sites)

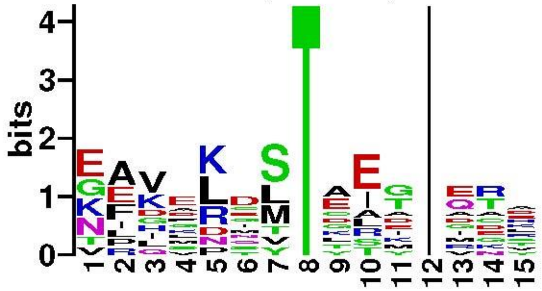

pXXXY (14 sites)

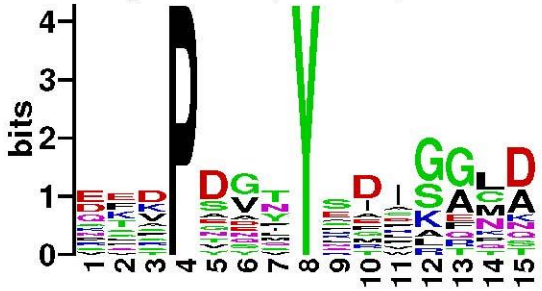

SXi (15 sites)

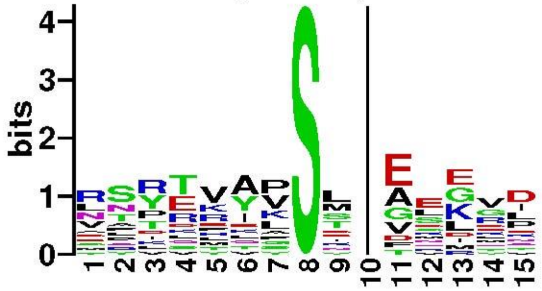

dXXXXXXS (15 sites)

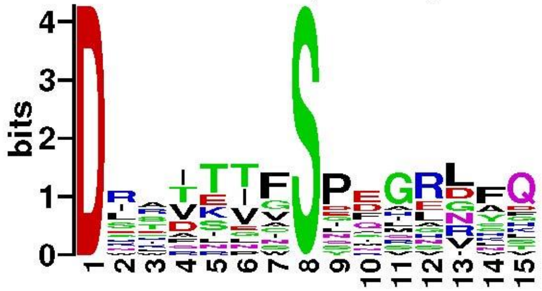

Supplement: Supplementary file 1 — Supplementary material 1 (PDF 2442 kb) [file 13238_2017_382_MOESM1_ESM.pdf]
